# Targeted next‐generation sequencing approach for molecular genetic diagnosis of hereditary colorectal cancer: Identification of a novel single nucleotide germline insertion in adenomatous polyposis coli gene causes familial adenomatous polyposis

**DOI:** 10.1002/mgg3.505

**Published:** 2018-12-06

**Authors:** Dan Wang, Shengyun Liang, Xipeng Zhang, Subrata Kumar Dey, Yuwei Li, Chen Xu, Yongjun Yu, Mingsen Li, Guoru Zhao, Zhao Zhang

**Affiliations:** ^1^ Department of Pathology Tianjin Medical University General Hospital Tianjin China; ^2^ Shenzhen Institutes of Advanced Technology Chinese Academy of Sciences Shenzhen China; ^3^ Shenzhen College of Advanced Technology University of Chinese Academy of Sciences Shenzhen China; ^4^ Department of Colorectal Surgery Tianjin Union Medical Center Tianjin China; ^5^ Department of Biotechnology, Centre for Genetic Studies, School of Biotechnology and Biological Sciences Maulana Abul Kalam Azad University of Technology (Formerly West Bengal University of Technology) Salt Lake City, Kolkata India

**Keywords:** *APC* gene, Chinese population, colorectal cancer, familial adenomatous polyposis, single nucleotide deletion, targeted next‐generation sequencing

## Abstract

**Background:**

Familial adenomatous polyposis (FAP) is an autosomal dominantly inherited disease which primarily manifested with developing adenomas or polyps in colon or rectum. It is caused by the germline mutations in adenomatous polyposis coli (*APC*) gene. Patients with FAP are usually manifested with “hundreds or even thousands” adenomas or polyps in colon or rectum. However, without proper clinical diagnosis and timely surgical interventions, colorectal adenomas, or polyps gradually increase in size and in numbers which finally leads to colorectal cancer (CRC) at the mean age of 36 years of the patient.

**Methods:**

In this study, we identified a family with FAP. In this family, FAP has been diagnosed clinically based on symptoms, medical test reports, and positive family history for three generations. In order to unveil the molecular genetic consequences underlying the disease phenotype, we performed next‐generation sequencing with a customized and designed panel of genes reported to be associated with hereditary CRC. The variant identified by next‐generation sequencing has been validated by Sanger sequencing.

**Results:**

A heterozygous novel insertion [c.3992_3993insA; p.Thr1332Asnfs*10] in exon 16 of *APC* gene has been identified. This novel insertion is cosegregated well with the FAP phenotype among all the affected members of this family. This mutation causes a frameshift by the formation of a premature stop codon which finally results in the formation of a truncated APC protein of 1,342 amino acids instead of the wild type APC protein of 2,843 amino acids. Hence, this is a *loss‐of‐function* mutation. This mutation was not found in unaffected family members or in normal control individuals.

**Conclusion:**

Our present study emphasizes the importance of a novel approach of the gene panel‐based high‐throughput sequencing technology for easy and rapid screening for patients with FAP or CRC which will help the clinician for follow‐up and management.

## 1 INTRODUCTION

Familial adenomatous polyposis (FAP) [MIM# 175100] is an autosomal dominantly inherited disease which primarily manifested with developing adenomas or polyps in colon or rectum. It is caused by the germline mutations in adenomatous polyposis coli (*APC*) gene [OMIM# 611731]. Patients with FAP are manifested with multiple polyps or adenomas in colon and rectum. FAP is very rare with an incidence of 3–10/100,000 worldwide. Approximately, FAP is accounting for 1% among all colorectal cancers cases (CRC) (Aretz, Genuardi, & Hes, 2013; Zhang et al., 2017). Patients with FAP gradually develop symptomatic CRS at the mean age of 40 years. However, timely, rapid, and accurate clinical diagnosis through genetic screening and early surgical interventions (proctocolectomy) could be a therapeutic measure for the patients with FAP (Marabelli et al., 2017; Yanus et al., 2018; Yu et al., 2018). In FAP patients, extracolonic manifestations are also reported rarely (Ghatak et al., 2017). The patients with classical FAP (CFAP) are characterized by early‐onset age with >100 colorectal polyps, whereas patients with attenuated FAP (AFAP) are presented with late‐onset age and less number (<100) of colorectal polyps (Khan, Lipsa, Arunachal, Ramadwar, & Sarin, 2017; Wachsmannova, Mego, Stevurkova, Zajac, & Ciernikova, 2017; Yedid et al., 2016).

Familial adenomatous polyposis is caused by the germline mutations of *APC* gene. It is a tumor suppressor gene, encoding APC protein with eight subdomains. APC protein is functioning in transcriptional regulation, cell movement, and cell death by regulating the β‐catenin protein level in cytoplasm (Spier et al., 2016; Zhang et al., 2016). Hence, mutated APC protein causes presence of high level of β‐catenin protein in cytoplasm which in turn causes loss of regulation in cell division and migration and finally develops CRC (Li et al., 2015; Liu et al., 2015; Papp et al., 2016). Mostly, frameshift, splicing, and intronic mutations were reported for *APC* gene associated with FAP (Lin et al., 2015; Stachler, Rinehart, Lindeman, Odze, & Srivastava, 2015). These three types of mutations always result in the formation of truncated APC protein. The clinical symptoms and onset age of FAP patients are also correlated well with the location and the type of mutations in the *APC* gene (Ashktorab et al., 2015; Patel et al., 2015).

Here, we have done a gene panel‐based high‐throughput targeted next‐generation sequencing and Sanger sequencing for the molecular genetic study of the members of this family. A novel heterozygous germline insertion [c.3992_3993insA; p.Thr1332Asnfs*10] in the exon 16 of the *APC* gene has been identified. This frameshift mutation is cosegregated well with the FAP phenotype among all the affected members in an autosomal dominant mode of inheritance.

## 2 MATERIALS AND METHODS

### 2.1 Ethical statement

We obtained written consent from all the participants in this study. The study plan and protocol has been approved by the Ethical Committee of the Tianjin Union Medical Center, China, in compliance with the Helsinki declaration.

### 2.2 Patients and pedigree

**Figure 1:**
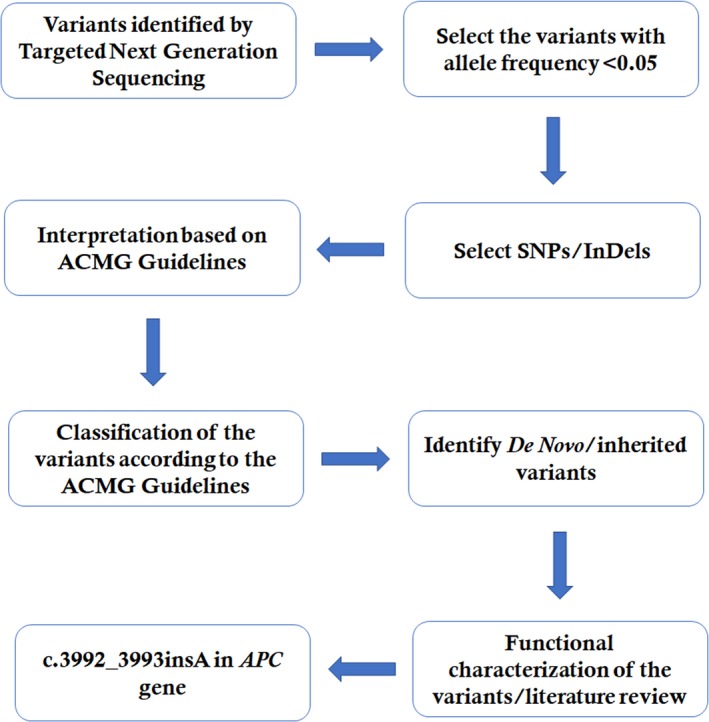
Data interpretation pipeline

Here, we identified and studied a family with FAP. The family is three generation with several members (Figure 1). Clinical diagnosis (colonoscopy and pathology) of FAP and treatment has been done in the Department of Colorectal Surgery, Tianjin Union Medical Center, 300121, China. The clinical diagnosis followed the standard or criteria for FAP patients based on (a) individuals with >100 polyps or adenomas in colon and rectum; and (b) individuals with minimum 20 polyps or adenomas in colon or rectum of the patients with a positive family history of FAP.

### 2.3 Targeted exome‐based next‐generation sequencing and variant identification

Proband's blood sample was collected, and genomic DNA was extracted. Proband's DNA samples obtained from the proband (III‐1) were sequenced using target exome‐based next‐generation sequencing. We use Roche NimbleGen's (Madison, USA) Sequence Capture Human Array to customize and designed a gene panel of 14 genes reported to be associated with LS or hereditary CRC. The total capture region containing 181 exons and flanking introns with 98,480 kb in length. These 14 genes are as follows: *APC*,* MLH1*,* MSH2*,* MSH6*,* PMS2*,* AXIN2*,* BMPR1A*,* EPCAM*,* MLH3*,* MUTYH*,* PMS1*,* PTEN*,* SMAD4*,* and STK11*. The average read per sample is 6,864,654 with 72% mapping of the target region. The average sequencing depth of the target area is 464.68% with 99.46% coverage. The quality control details for this targeted NGS have been given in Table 1. Data interpretation pipeline is described in Figure 1.

**Table 1:** Quality control result of targeted next‐generation sequencing

Raw reads (mapped to hg19)	9,149,918
Raw data yield (Mb)	823.49
Reads mapped to target region	5,820,840; 63.62%
Reads mapped to flanked 100 bp region	6,064,484; 66.28%
Data mapped to target region (Mb)	457.79; 55.59%
Data mapped to flanked 100 bp region (Mb)	476.72; 57.89%
Length of target region	838,543
Length of flanked 100 bp region	993,609
Number of covered bases on target region	830,733
Coverage of target region	99.07%
Number of covered bases on flanked 100 bp region	983,414
Coverage of flanked 100 bp region	98.97%
Average sequencing depth of target region	545.94
Average sequencing depth of flanked 100 bp region	479.79
Fraction of target region covered with at least 4×	829,222; 98.89%
Fraction of target region covered with at least 10×	828,386; 98.79%
Fraction of target region covered with at least 20×	826,937; 98.62%
Fraction of target region covered with at least 30×	825,582; 98.45%
Fraction of flanking region covered with at least 4×	981,373; 98.77%
Fraction of flanking region covered with at least 10×	980,315; 98.66%
Fraction of flanking region covered with at least 20×	978,150; 98.44%
Fraction of flanking region covered with at least 30×	975,554; 98.18%

The procedure for the preparation of libraries was consistent with standard operating protocols published previously (Wei et al., 2014).

### 2.4 Confirmation of the novel deletion mutation by Sanger sequence

To validate true positive of the mutation, Sanger sequencing was performed.

The heterozygous novel mutations identified through targeted next‐generation sequencing were verified through Sanger sequencing using the primers: F1 5′‐TTTAGGAGTGATTTACGGGC‐3′, R1 5′‐GTTTGTGGGAATCCGCCAAGTA‐3′. The reference sequence NM_000038 of *APC* was used.

## 3 RESULTS

### 3.1 Family recruitment and clinical examination

In this family, among 14 members, four individuals were reported with FAP. Proband (III‐1) has been suffering from FAP, and another three individuals died due to CRC (I‐2, II‐2, and II‐3; Figure 1). Another 10 individuals are unaffected.

The proband (III‐1) was a 36‐year‐old female presented with a clinical history of blood in the stool and mild anemia for last 2 years. She also had the history of diarrhea for more than half a year. The proband was admitted to the hospital for treatment. The proband was weak with poor appetite but no nausea and vomiting were found. The proband had slight weight loss. The proband had no fever and also had normal sleeping.

These family members have been clinically diagnosed based on the colonoscopy, pathological test, and segregation analysis. In Table 2, the detailed clinical features of all the family members are described.

**Table 2:** Clinical characteristics of all the affected and unaffected family members found in our study

ID	Sex	WT/MT	Present age (years)	Onset age (years)	Clinical symptoms	No. of colorectal adenomas or polyps
I‐1	M	—	—	—	—	—
I‐2	F	—	Die (50)	—	Intestinal cancer	Unknown
II‐1	M	—		—		
II‐2	F	—	Die (41)	—	FAP, cancerous	Unknown
II‐3	M	—	Die (40)	—	FAP, cancerous	>1,000
II‐4	F	—		—	—	—
II‐5	M	—	64	—	—	Colonoscopy (−)
II‐6	F	—		—	—	—
II‐7	M	—		—	—	—
II‐8	F	—	62	—	—	Unknown
III‐1	F	MT	36	36	Intermittent diarrhea and stomach ache/bloody stools/FAP/cancerous	>1,000
III‐2	M	—	36	—	—	—
III‐3	F	—	36	—	—	—
III‐4	F	—	34	—	—	—

Note

FAP: familial adenomatous polyposis; WT: wild type; MT: mutant.

### 3.2 Colonoscopy

**Figure 2:**
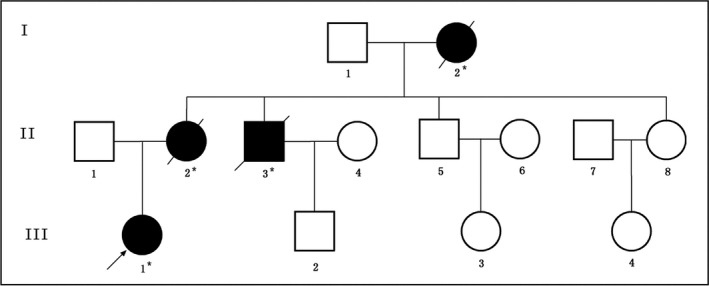
Pedigree structure of the Chinese family with familial adenomatous polyposis (FAP). Family members with FAP are indicated with Shading. Squares and circles denoted males and females, respectively. Roman numerals indicate generations. Arrow indicates the proband (III‐1)

The colonoscopy of the proband (III‐1) showed that approximately 1,000 polyps and local bulge type tumor in the colon, the maximum diameter was about 3 cm (Figure 2a). The center of the tumor showed ulcer and considered FAP and carcinogenesis (Figure 2a). The colonoscopy of the unaffected individual (II‐5) showed no abnormality (Figure 2b). After total colorectal resection, the colon showed about a thousand polyps. The maximum diameter was about 3 cm, hard (Figure 2c).

### 3.3 Pathology

**Figure 3:**
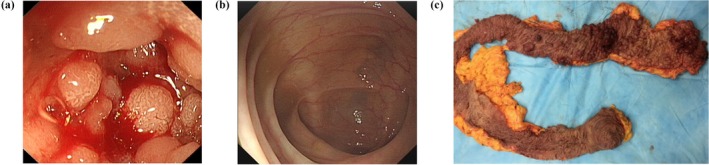
Clinical description. Colonoscopy of the proband (III‐1): (a) The colonoscopy showed about a thousand polyps and local bulge type tumor in the colon; the maximum diameter was about 3 cm. The center of the tumor showed ulcer, considered familial adenomatous polyposis and carcinogenesis. (b) Normal Colon from unaffected individual (II‐5). (c) After total colorectal resection, the colon showed about a thousand polyps. The maximum diameter was about 3 cm hard

Pathology of the proband (III‐11) identified with moderately differentiated adenocarcinoma (Figure 3a) and chronic mucosal inflammation (Figure 3b).

On the basis of colonoscopy and pathology result, we recommend her to perform a total colon resection. She had undertaken a total colon resection under general anesthesia by laparoscopic method. Prevention from infection, prevention of bleeding, rehydration, nutrition, and other follow‐up treatment was performed after surgery.

### 3.4 Identification and characterization of candidate mutation

**Figure 4:**
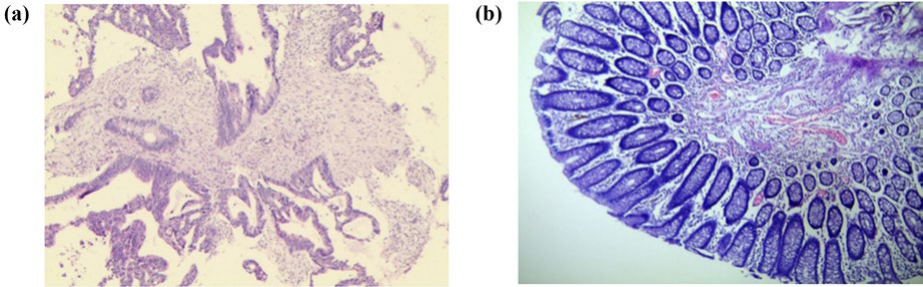
Clinical description. Pathology of the proband (III‐1): (a) The pathology showed moderately differentiated adenocarcinoma. (b) Chronic mucosal inflammation

A novel heterozygous germline insertion; [c.3992_3993insA; p.Thr1332Asnfs*10] in exon 16 of *APC* gene [NCBI Reference sequence NM_000038.3] in the proband (III‐1) was identified. Sanger sequencing confirmed that this mutation is cosegregated well with the FAP phenotype among all the affected family members but not found in unaffected family members as well as in the normal control (Figure 4).

## 4 DISCUSSION

**Figure 5:**
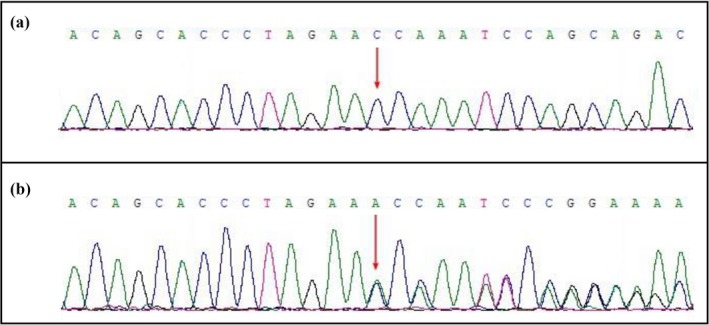
Confirmation of the novel insertion mutation by Sanger sequence. (a) Sanger sequencing of unaffected family members. (b) A novel heterozygous germline insertion [c.3992_3993insA; p.Thr1332Asnfs*10] in the exon 16 of the *APC* gene has been identified in the proband and among all the affected family members

Here, we identified a heterozygous novel mutation [c.3992_3993insA; p.Thr1332Asnfs*10] in exon 16 of the *APC* gene in the proband (III‐1) in this three generation Chinese family (Figure 5). This heterozygous novel insertion of *APC* gene leads to frameshift by a premature stop codon which finally results in the formation of a truncated APC protein of 1,342 amino acids, almost half a length compared with the wild type APC protein consisting of 2,417 amino acid. Therefore, this mutation is a *loss‐of‐function* mutation causing disease following the haploinsufficiency. This mutation is not present in both 1,000 genome database and ExAC database. This mutation is a likely pathogenic mutation based on ACMG guidelines (Richards et al., 2015). In this present case, clinical diagnosis of FAP has been confirmed based on clinical symptoms (adenomas or polyps in colon and rectum) of the proband and the autosomal dominance inheritance pattern. In this study, the proband was not identified with extracolonic manifestations.

Till date, germline mutations in *APC* genes are accounting for the major cause of hereditary CRS. In Chinese population, till now, 33 germline single nucleotide insertions of *APC* gene have been reported (LOVD database). All these *APC* germline single nucleotide insertions lead to frameshift which followed by the formation of a truncated APC protein (Cao, Weng Eu, Seow‐Choen, Zao, & Yean Cheah, 2000; Huang, Zheng, Jin, & Zhang, 2004; Liu et al., 2007; Pang et al., 2001). Among these 33 germline single nucleotide insertions, 21 are located at exon 16, the longest exon and the mutation hotspot in *APC* gene.

Presently, targeted next‐generation sequencing is one of the best platforms for genetic screening for cancer predisposition. Targeted next‐generation sequencing provides easy and high‐throughput genetic screening for the patients with FAP for easy and early clinical diagnosis and timely treatment, follow‐up and management (Bosdet et al., 2013; Feliubadaló et al., 2013). In order to solve the problem of clinical diagnosis and genetic molecular analysis of FAP patients for their extreme genetic heterogeneity and overlapping clinical manifestations, targeted gene panel‐based next‐generation sequencing technology provides a more cost‐effective, less time‐consuming, comprehensive, and detailed information rather than whole exome sequencing (Couch et al., 2015; De Leeneer et al., 2015). Hence, sequencing with a gene panel is more significant for routine genetic screening for hereditary cancer patients (Kurian et al., 2014; Walsh et al., 2010). In addition to the interpretation and validation of targeted NGS‐driven genetic screening, standard guidelines with recommendations have been published (Desmond et al., 2015; Gargis et al., 2012; Robson et al., 2015). However, bioinformatic analysis pipeline and interpretation of variants are very important for understanding the candidate genes and pathogenic mutations.

## AVAILABILITY OF DATA AND MATERIALS

The datasets used and/or analyzed during the current study are available from the corresponding author on reasonable request.

## CONFLICT OF INTERESTS

The authors state no conflict of interests.

## AUTHORS’ CONTRIBUTIONS

DW, SL, XZ, SKD, and ZZ designed the study and drafted the manuscript. XZ, YL, CX, YY, ML, and GZ acquired and interpreted the data. All authors read and approved the final manuscript.
